# The role of physical therapists in the early detection of skin melanoma: insights from an anonymous survey

**DOI:** 10.3389/fmed.2024.1436206

**Published:** 2024-08-06

**Authors:** Bar Arouch, Michal Elboim-Gabyzon

**Affiliations:** ^1^Department of Physical Therapy, School of Social Welfare and Health Sciences, University of Haifa, Haifa, Israel; ^2^Clalit Health Services, Holon Branch, Holon, Israel

**Keywords:** skin melanoma, physical therapy, health promotion, detection, education

## Abstract

**Introduction:**

Skin melanoma is the most severe form of skin cancer. Recent years have seen an increase in melanoma incidence. Melanomas often appear on the back, a hidden area, leading to late diagnoses. Physical therapists, who frequently treat patients with lower back pain, could play a crucial role in early melanoma detection.

**Methods:**

An anonymous online survey was conducted among 254 Israeli physical therapists to assess their perspectives on melanoma detection, their knowledge in identifying suspicious lesions, and their referral patterns. The survey included sections on demographics, professional perspectives, melanoma knowledge, personal or family experiences with melanoma, and referral frequency for dermatological evaluation.

**Results:**

The survey revealed that 75.2% of physical therapists viewed melanoma detection as part of their professional duties, yet 59.1% reported insufficient knowledge in identifying suspicious lesions. Despite this, 94.1% expressed a desire to improve their knowledge. However, only 44.1% actively referred patients with suspicious lesions to dermatologists. There was a significant positive correlation between professional experience and referral rates (*p*-value < 0.001), indicating that more experienced therapists were more likely to refer patients.

**Discussion:**

The study highlights the critical need for incorporating melanoma detection training into physical therapy education and professional development. Enhancing physical therapists’ knowledge and skills in this area could improve early detection and patient outcomes. Despite the potential role of physical therapists in melanoma detection, current training programs lack emphasis on this aspect, underscoring the importance of revising educational curricula to include skin cancer detection techniques.

**Conclusion:**

The findings suggest that while physical therapists recognize their role in melanoma detection, there is a significant knowledge gap. Addressing this through targeted education and training could enhance early detection efforts and improve patient care.

## 1 Introduction

Skin cancer is the most prevalent type of cancer, classified into: non-melanoma skin cancer (NMSC) and melanoma (1). NMSC progresses slowly and responds well to early treatment, often leading to successful outcomes. Melanoma poses greater challenges, including difficulty in early detection, higher metastasis rates, and a significantly higher fatality rate compared to NMSC. Melanoma initially manifests as firm, asymmetrical spots of varying colors, which may change color, rise from the skin, or bleed as the disease progresses (1, 2). The American Joint Committee (AJCC) TNM staging system classifies melanoma by tumor size (T), lymph node involvement (N), and metastasis (M), ranging from stage 0 to IV. Key factors include tumor thickness, ulceration, mitotic rate, and metastasis, guiding prognosis and treatment decisions (3). Typical melanoma lesion locations differ by gender, appearing on the back and neck in men, and on the limbs and back in women (2). The worldwide occurrence of melanoma and the median age at which it is diagnosed remain consistent across different regions (3). In 2019, 1,818 new cases of melanoma were recorded in Israel, resulting in 213 deaths, with advanced melanoma (stages 3 and 4) diagnosis ages averaging 66.7 years for male and 63.9 for female (3).

Increased melanoma diagnoses in Israel mirrors global trends. A phenomenon attributed to an actual increase in number of cases coupled with improvements in detection methodologies (3). This trend is reflective of patterns observed in other nations, including Australia, France, and Colombia, with melanoma rates predicted to rise by over 50% by 2040 (4). Early detection is crucial due to the lack of effective treatments for advanced melanoma. Timely intervention, mainly through surgical excision, is vital for improving patient outcomes, reducing mortality, and enhancing the quality of life. A previous study in the United States (2016) (5) demonstrated that over 53% of initial melanoma detections were made by the patients themselves, underscoring the importance of self-awareness. Dermatologists identified 20% during routine examinations; family and friends 16%; and other healthcare professionals, including doctors, nurses, pharmacists, podiatrists, and physical therapists, 11%. Patients who self-diagnosed often sought medical help at more advanced stages, resulting in poorer outcomes compared to those identified by dermatologists (5). Additionally, melanomas in hard-to-see areas were often diagnosed at later stages. Characteristics commonly observed in individuals diagnosed at later stages encompass elderly males aged 70 and above, those living alone, and individuals with lower levels of education (6). This underscores the vital role of healthcare professionals, including physical therapist, in early diagnosis, especially for lesions in hard-to-see areas (5). The high incidence and late-stage diagnoses of melanoma underscore the need for urgent attention, particularly among elderly males, those living alone, and individuals with lower education levels (3, 7).

Incorporating physical therapists into the early detection process of skin melanoma during physical therapy evaluations is logical, especially given the high prevalence of lower back pain among seniors (ages 60–102 years), affecting 75% of this age group (8). Many seniors seek community-based physical therapy, where routine physical examinations can reveal hard-to-see areas like the back, giving physical therapists a unique opportunity to spot potential signs of melanoma. Despite this potential, melanoma detections by physical therapists in clinical practices are notably low (5, 9), likely due to insufficient knowledge and expertise in identifying skin pathologies, highlighting the need for advanced training in skin cancer detection (4–6, 9–12).

Current training programs for healthcare practitioners, including physical therapists, frequently neglect the critical role in early skin cancer detection (6). There is a widely recognized need to incorporate skin cancer detection training into healthcare education, with dermatologists educating on early diagnosis (6, 10, 13). A comprehensive review of worldwide of physical therapy curricula, shows significant gaps in teaching melanoma detection (12, 14). A 2020 Irish survey of 192 radiologists, physical therapists, and hospital practitioners revealed that 65% of the respondents had no training on skin cancer detection, 8% encountered it during undergraduate studies, and 16% post-graduation. Remarkably, 95% of the participants expressed a strong interest to expand their knowledge on early skin cancer detection. While 76% noticed suspicious lesions, 44% did not inform patients, 27% consulted a physician, and 29% advised both self-examination and professional consultation (12).

To date, no comparable surveys have focused specifically on physical therapists. This current study is crucial for investigating physical therapists’ perceptions of their role in detecting skin melanoma and evaluating their knowledge of detection techniques. Understanding these viewpoints is essential for improving training programs, and developing continuing education courses.

The current research objectives are as follows:

1. To investigate whether physical therapists perceive detecting skin melanoma within their practice role.
2. To assess whether they possess the knowledge necessary for identifying suspicious skin lesion.
3. To explore their willingness to acquire knowledge about skin melanoma.
4. To evaluate their referral rate of patients with suspected skin lesions for further assessment by dermatologists.
5. To investigate the correlation between participants’ personal and professional characteristics and the frequency of patient referrals.

## 2 Materials and methods

The study received ethical approval from the Ethics Committee of the Faculty of Health Sciences and Welfare at the University of Haifa (approval number 201/23). An anonymous online survey was created and distributed via the Qualtrics platform between 12 June 2023, and 16 July 2023. The survey was distributed through multiple channels to ensure wide participation. It was advertised in the newsletter of the Association for the Advancement of Physical Therapy in Israel, posted in social media groups for physical therapists, and shared with clinical instructors. Additionally, the survey reached potential participants through word-of-mouth and personal networks of the research team. Eligibility for participation in this study was defined as follows: qualified physical therapist employed across various healthcare settings, such as acute care departments, rehabilitation centers, private practices, and public health facilities. The exclusion criterion was physical therapist whose primary focus is child development. The survey was designed to be concise, taking approximately ten minutes to complete.

The survey included five sections: (a) socio-demographic and professional information; (b) physical therapist’ perspectives on their role in skin melanoma detection; (c) existing knowledge about skin melanoma, including signs, diagnostic methods, and interest in additional learning; (d) personal or familial experiences with early skin melanoma detection; (e) frequency of referrals for dermatological evaluation in cases of suspicious lesions to rule out or confirm skin melanoma.

The survey specifically evaluated participants’ knowledge of the scale used as the melanoma detection tools. This scale, termed ABCDE scale, was developed by the American Academy of Dermatology and is globally acknowledged as the gold standard for early melanoma detection, and is widely implemented, including in Israel (15, 16). This tool aims to simplify the early diagnosis process for both medical professionals and individuals, detailing five primary signs indicative of melanoma within skin lesions (16). Detection of even a single characteristic warrants a comprehensive examination by a dermatologist. The criteria include: asymmetry, where the lesion doesn’t match on both sides; Border, characterized by uneven or vague edges; Color, with the lesion displaying a variety of shades, either of the same color or different colors; Diameter, where the lesion is larger than 6 mm, akin to a pencil eraser’s size; and Evolving, signifying any change in the lesion’s appearance. The efficacy of the ABCDE criteria, based on psychometric analysis, demonstrates an 89.3% sensitivity and a 65.3% specificity when two signs are detected. However, with three identified signs, while sensitivity falls to 65.5%, specificity climbs to 81% (17).

### 2.1 Statistical analysis

To evaluate the distribution of answers among the variables of the survey, frequency tables were employed. This method was further enhanced by the use of Chi-Squared tests to compare the variance in responses for each question. For questions with fewer than three options, the Fisher test was applied, owing to its effectiveness in analyzing scenarios with limited data. This is because the Chi-Squared test may not be as accurate in cases of sparse data, rendering the Fisher test a preferable alternative.

## 3 Results

### 3.1 Participant characteristics

#### 3.1.1 A. Sociodemographic and professional characteristics of the respondents

The survey included 254 practicing physical therapists from diverse sociodemographic and professional settings. Among these participants, 67 were male (26.4%), while 187 (73.6%) were female. The average age of the respondents was 40.6 years (standard deviation of 10.5). Detailed data about their educational qualifications, workplace, fields of practice, and length of professional experience are provided in Table 1.

**TABLE 1:** Sociodemographic and professional characteristics of the participants (*N* = 254).

Category	Count (percentage)
**Educational qualification**	
Bachelor’s degree in physical therapy	177 (69.7%)
Additional bachelor’s degree	12 (4.7%)
Master’s degree in physical therapy	40 (15.7%)
Additional master’s degree	22 (8.7%)
Doctorate in physical therapy	2 (0.8%)
Other doctoral degree	1 (0.4%)
**Geographical origin of education**	
Israel	247 (97.2%)
United States	1 (0.4%)
Europe	1 (0.4%)
Other regions	5 (2%)
**Primary employment setting**	
Hospital	53 (20.9%)
Public clinic	97 (38.2%)
Private clinic	77 (30.3%)
Rehabilitation center	26 (10.2%)
Home-based services	43 (17%)
Athletic teams	7 (2.8%)
Nursing facilities	4 (1.6%)
Other workplaces	25 (9.8%)
**Years of professional experience**	
0–5 years	80 (31.5%)
5–16 years	102 (40.2%)
Over 16 years	72 (28.3%)

Due to the option for participants to select more than one primary work setting, the total percentage may exceed 100%.

#### 3.1.2 B. Physical therapists’ perspectives on their role in early detection of skin melanoma within physical therapy practice

**FIGURE 1:**
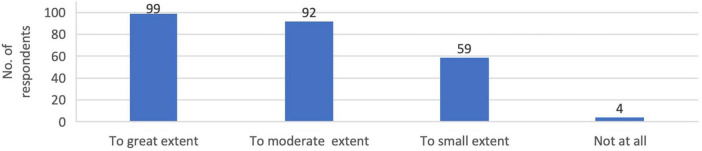
Physical therapists’ perception of their role in detecting skin melanoma (*N* = 254).

A substantial portion of the survey participants (75.2%) recognized the detection of suspicious skin lesions as within a physical therapist’s professional scope, with 39 and 36.2% expressing strong or moderate with this statement. Remarkably, only a tiny minority (1.6%) did not consider lesion detection to be within the physical therapist’s responsibilities. These results are depicted in Figure 1.

#### 3.1.3 C. Assessing participants’ knowledge of melanoma

A notable proportion of the respondents, 59.1% (150 individuals), acknowledged their inability to accurately identify lesions indicative of skin cancer. Conversely, 40.9% (104 individuals) affirmed their capability in this regard, citing various sources for their expertise. The most common source was personal experience, cited by 32.7% of those with proficiency, followed by professional experience in the field, which accounted for 21.2%. Comprehensive details are provided in Table 2.

**TABLE 2:** Sources of knowledge for identifying suspected skin lesions.

Knowledge source	Count (percentage)
Bachelor’s degree in physical therapy	16 (15.4%)
Master’s degree in physical therapy	2 (1.9%)
Specialized postgraduate courses	15 (14.4%)
Experience from professional practice	22 (21.2%)
Direct personal experience (history)	34 (32.7%)
Other sources	15 (14.4%)

Participants were allowed to select more than one source of knowledge, leading to a cumulative percentage that may exceed 100%.

A significant portion of the respondents (77.2%), were not acquainted with the ABCDE tool. Meanwhile, 17.3% (44 individuals) had some knowledge of it, and a mere 5.5% (14 individuals) had a comprehensive understanding.

#### 3.1.4 D. Interest in learning about skin cancer detection techniques

An overwhelming 94.1% of participants showed a strong interest in learning methods for early detection of skin cancer among patients, with only 5.9% of the therapists, showing no interest in expanding their knowledge on skin cancer detection.

#### 3.1.5 E. Early personal/family history exposure of skin melanoma

A vast majority of the participants, 82.3% (209 individuals), reported no personal or familial diagnoses of skin cancer.

#### 3.1.6 F. Patient referrals for dermatological assessments

Nearly half of the respondents, 44.1% (112 individuals), stated they had not referred any patients for further dermatological assessments. A smaller portion, 32.3% (82 individuals), had referred fewer than five patients, whereas 23.6% (60 individuals) had referred more than five patients for additional evaluation by a dermatologist.

### 3.2 Correlation between professional experience and referral rates

There was a significant positive correlation between a physical therapists’ years of professional experience and the frequency of referring patients for dermatological consultations (*p*-value < 0.001). Only 5% of those with 0–5 years of experience referred more than five patients, compared to 24% of those with 6–15 years, and 43% with over 16 years of experience. A Chi-Square test (4 degrees of freedom, value 37.6) further supports these observations, with additional information presented in Table 3.

**TABLE 3:** Referral rates to dermatologists based on physical therapist’ experience and knowledge (*N* = 254).

Category	Total	Not referred (%)	Referred < 5 patients (%)	Referred > 5 patients (%)
**Years of experience**				
0–5 years	80	52 (65%)	24 (30%)	4 (5%)
6–15 years	102	42 (41.2%)	35 (34.3%)	25 (24.5%)
Over 16 years	72	18 (25%)	23 (31.9%)	31 (43.1%)
Total	254	112 (44.1%)	82 (32.3%)	60 (23.6%)
**Existing knowledge**				
Presence of prior knowledge	150	86 (57.3%)	44 (29.3%)	20 (13.3%)
Without prior knowledge	104	26 (25%)	38 (36.5%)	40 (38.5%)
Total	254	112 (44.1%)	82 (32.3%)	60 (23.6%)

A significant positive correlation (*p*-value < 0.001) was found between the number of patient referrals to dermatologists and the physical therapist’ self-reported knowledge in identifying skin lesions, as confirmed by Chi-Square analysis (2 degrees of freedom, value 32). Further details can be found in Table 3.

Moreover, the study identified no significant differences in referral rates to dermatologists when comparing gender or employment in the public versus private sector. Additionally, there was no significant differences in referral incidence among physical therapist with a personal or familial history of skin melanoma compared to those without such backgrounds, as depicted in the comparative analyses outlined in Table 4.

**TABLE 4:** Referral rates to dermatologists based on workplace, gender, and skin condition history (*N* = 254).

Category	Total	Referred < 5 patients (%)	Referred > 5 patients (%)	Not referred (%)
**Workplace set**				
Public clinic	89	32 (36%)	27 (30.3%)	30 (33.7%)
Private clinic	60	26 (43.3%)	20 (33.3%)	14 (23.3%)
**Personal or family history of skin issues**				
No	209	65 (31.1%)	48 (23%)	96 (45.9%)
Yes	45	17 (37.8%)	12 (26.7%)	16 (35.6%)
**Gender**				
Men	67	21 (31.3%)	16 (23.9%)	30 (44.8%)
Women	187	61 (32.6%)	44 (23.5%)	82 (43.8%)

Regarding the relationship between professional experience and a personal or family history of melanoma, no significant association was observed (*p*-value > 0.05), according to the Chi-Square results (2 degrees of freedom, value 1.41); these results are detailed in Table 5.

**TABLE 5:** Frequency of personal or family skin condition diagnoses by years of experience (*N* = 254).

Years of experience	Total	Diagnosis present (yes) (%)	Diagnosis absent (no) (%)
0–5 years	80	13 (16.3%)	67 (83.6%)
6–15 years	102	16 (15.7%)	86 (84.3%)
Over 16 years	72	16 (22.2%)	56 (77.8%)
Total	254	45	209

## 4 Discussion

This study assessed physical therapists’ knowledge and perceived role in early melanoma detection during clinical interactions, and their interest in expanding this knowledge. It also examined how participant characteristics influenced patient referral rates for further medical evaluation.

One objective was to assess physical therapist’ views on their role as initial detectors of skin melanoma within their practice scope. A significant majority (75.2%) agreed that identifying suspicious skin lesions aligns with their professional role and responsibility. This consensus reflects broader acceptance found in the literature of physical therapist’ roles in skin cancer awareness and education (1, 4–6, 9–12). Dundas (10) highlights the importance of physical therapy in patient education on routine skin examination, in line with the American Cancer Society’s monthly self-examination recommendation. This perspective is reinforced by research among massage therapists (18), highlighting their potential in early melanoma detection due to frequent skin exposure during treatments (1, 9). Although not explicitly listed in their professional scope, early detection of skin conditions falls under health promotion and disease prevention, crucial areas recognized by the American Physical Therapy Association (APTA) (19). The ethical code for Israeli physical therapy supports the value of health promotion and of exploring new public health avenues related to their expertise (20). Despite this, integrating skin lesion detection into healthcare curricula remains limited globally, despite the rising incidence of melanoma (3, 4). Studies from New Zealand, Australia, and France show promising results for other healthcare professionals (nurses and family doctors) in skin cancer detection after minimal training, suggesting similar training for physical therapists could enhance their knowledge and professional development, improving public health and patient care (13, 21).

Findings reveal a notable knowledge gap among physical therapist: 59.1% reported a lack of knowledge in identifying potential cancerous skin lesions, with most knowledge gained independently or through practical experience (13.4 and 8.7%, respectively). Few reported learning this in their undergraduate/postgraduate education, or in post-degree training (6.3, 0.8, and 5.9%, respectively). Aligning these findings with previous studies is challenging since most focus on general practitioners (21–23). To our knowledge, only one study explored this topic among healthcare professionals, including physical therapists (12), and another among massage therapists (18). These studies align with the current findings, showing a lack of training during professional education. Quinlan et al. (12), found that only 8% of healthcare professionals learned about identifying and referring suspect skin lesions during their undergraduate curriculum, 16% post-graduation, and 76% had no exposure at all (12). Although Quinlan et al. (12) assessed knowledge levels among physical therapists, they were not the sole focus; the study included a broader range of healthcare professionals (48% radiologists, 33% physical therapist, and 19% practicing physicians). The current study and Quinlan et al. (12) study suggest that healthcare professionals, particularly physical therapists, often lack training in primary detection of skin lesions. This gap may be due to the belief within academic circles and continuing education bodies that this subject fall outside the core domain of these professions. Surprisingly, over half (60%) of 262 US massage therapists were educated on skin cancer detection during training (18). Despite similarities between the professions, physical therapy differs in educational requirements, licensure, practice scope, and therapeutic goals. It’s striking that physical therapy, especially in warmer regions with heightened awareness of health risks, has not yet integrated early melanoma detection into its educational framework (24).

Survey participants were queried about their familiarity with the ABCDE detection tool, a gold standard and a quick screening method for skin cancer (10). Our findings reveal that a significant majority of physical therapists (77.2%) are unfamiliar with this tool. While no prior studies focus specifically on physical therapy, research in Brazil (2020) shows a similar trend among family physicians, with 64.6% unfamiliar with the ABCDE method (22). This suggests a broader knowledge deficit concerning the ABCDE tool across medical disciplines, despite evidence supporting its effectiveness (15, 16).

The current survey reveals that nearly half of the respondents (44.1%) did not refer patients to a dermatologist for further evaluation. This aligns with Quinlan et al.’s (12), which found that while over three-quarters of healthcare professionals, including physical therapists, noticed suspicious skin lesions on patients, 44% did not inform their patients. Similarly, Avilés-Izquierdo et al. (5) found that physical therapists, among other non-dermatologist providers, identified only 11% of 784 melanoma cases. Avilés-Izquierdo et al. (5) noted obstacles for general practitioners, such limited time and insufficient exposure, particularly for lesions in hard-to-see areas. The current study aimed to investigate factors influencing physical therapists’ decisions to refer patients for further dermatological evaluation. Our finding revealed a significant positive correlation between a physical therapist’s years of experience and the rate of dermatologist referrals (*p*-value < 0.001). This correlation may be attributed to the cumulative knowledge and practical skills gained over time. No other studies have investigated this specific relationship. Additionally, we found a significant correlation between referral rates and physical therapist’ self-reported knowledge in identifying skin lesions (*p*-value < 0.001). Those knowledgeable in this area were more likely to refer patients. This observation aligns with prior research indicating that knowledge is crucial in the referral process and early melanoma detection among physical therapists (1, 5, 6, 9, 10, 12). Another potential reason for the observed link between greater experience and increased referral rates could be the heightened likelihood of encountering melanoma personally or within the family as one ages. This theory is supported by Campbell et al.’s (18) study, which found that massage therapists who engaged in annual self-screenings for skin lesions were significantly more diligent in examining their patients (*p*-value < 0.001). Similarly, Avilés-Izquierdo et al. (5) noted that individuals with personal or familial histories of melanoma were more proactive in early detection. However, our study did not confirm a link between personal or familial melanoma experiences and referral rates (Chi-Square results: 2 degrees of freedom, odds ratio of 1.41, *p*-value > 0.05). This discrepancy may be due to the limited number of participants (17.7%) reporting such histories. Further research with larger cohorts is necessary to explore this hypothesis among physical therapist more thoroughly. This study did not find statistically significant differences in dermatologist referrals or eagerness to learn more about melanoma detection tools between male and female participants. Comparing these findings with previous studies on healthcare professionals is challenging due to the limited existing research. The initial hypothesis, inspired by Avilés-Izquierdo et al. (5), suggested higher referral rates among women, who were more likely to diagnose melanoma in their spouses and within their personal networks. However, the hypothesis was not supported, despite the larger female representation in this study (73.6% women, 26.4% men). This discrepancy may be due to cultural differences between participant groups in the current study and the US-based research (5).

Several limitations of this study should be acknowledged. The reliance on convenience sampling may not fully capture the diversity within Israel’s physical therapy population, potentially affecting representativeness. The use of self-reported data, especially for recalling where knowledge about melanoma detection was acquired, introduces the risk of memory bias. Closed-ended questions about the source of knowledge did not allow for detailed insights, particularly for those selecting “other.” Addressing these limitations in future studies could enhance understanding of physical therapists’ role in melanoma detection and improve their training and clinical practices.

## 5 Conclusion

The survey of 254 physical therapists revealed that while 75.2% recognize their role in melanoma detection, 59.1% noted a lack of training in their education. Only 44.1% had referred patients to dermatologists, yet 94.1% are eager to learn more about detection. Health promotion, a key part of physical therapy, underscores the need for awareness and training in melanoma detection, suggesting that professional organizations and education programs play critical roles in enhancing skills for better patient outcomes.

## Data availability statement

The raw data supporting the conclusions of this article will be made available by the authors, without undue reservation.

## Ethics statement

The studies involving humans were approved by the Ethics Committee of the Faculty of Health Sciences and Welfare at the University of Haifa (approval number 201/23). The studies were conducted in accordance with the local legislation and institutional requirements. The participants provided their written informed consent to participate in this study.

## Author contributions

BA: Conceptualization, Data curation, Formal analysis, Investigation, Methodology, Project administration, Resources, Software, Validation, Visualization, Writing – original draft, Writing – review & editing. ME-G: Conceptualization, Data curation, Formal analysis, Investigation, Methodology, Resources, Software, Supervision, Validation, Visualization, Writing – original draft, Writing – review & editing.
